# Immunolipid magnetic bead-based circulating tumor cell sorting: a novel approach for pathological staging of colorectal cancer

**DOI:** 10.3389/fonc.2024.1531972

**Published:** 2025-01-24

**Authors:** Qingyan Deng, Weidong Li, Yueming Huang, Haitao Wang, Xinhao Zhou, Zhifen Guan, Bohao Cheng, Yao Wang

**Affiliations:** Department of Gastrointestinal Surgery, Zhongshan People’s Hospital, Zhongshan, Guangdong, China

**Keywords:** colorectal cancer, NGS sequencing, immunolipid magnetic bead, circulating tumor cells, mutations in tumor tissue

## Abstract

**Objective:**

This study aimed to assess whether circulating tumor cells (CTCs) from colorectal cancer (CRC) could be used as an alternative to tissue samples for genetic mutation testing, overcoming the challenge of difficult tumor tissue acquisition.

**Methods:**

We developed an immunolipid magnetic bead (IMB) system modified with antibodies against epithelial cell adhesion molecule (EpCAM) and vimentin to efficiently separate CTCs. We prepared EpCAM-modified IMBs (Ep-IMBs) and vimentin-modified IMBs (Vi-IMBs). The separation efficiency of the system was evaluated via *in vitro* experiments and by capturing and counting CTCs in blood samples from 23 CRC patients and 20 healthy controls. Hotspot mutations in patient tissue samples were identified via next-generation sequencing (NGS), whereas mutations in blood CTCs were detected via Sanger sequencing. The concordance between hotspot mutations in tumor tissue and blood CTCs was analyzed.

**Results:**

The CTC sorting system exhibited good dispersion, stability, and low cytotoxicity, with a specificity of 90.54% and a sensitivity of 89.07%. CRC patients had an average of 8.39 CTCs per 7.5 mL of blood, whereas healthy controls had 0.09 per 7.5 mL of blood. The consistency of gene mutations was as follows: *TP53* (91.31%), *PIK3CA* (76.00%), *KRAS* (85.36%), *BRAF* (51.00%), *APC* (65.67%), and *EGFR* (74.00%), with an overall gene mutation consistency of 85.06%.

**Conclusion:**

Our CTC sorting system, which is based on Ep-IMBs and Vi-IMBs, effectively captures CTCs in the peripheral blood of CRC patients and enables clinical hotspot gene mutation testing via these enriched CTCs. This system partially solves the problem of difficult tumor tissue sample collection and provides a reference for gene mutation testing in early diagnosis, therapeutic efficacy evaluation, prognosis assessment, and minimal metastasis detection in CRC patients, showing significant potential for clinical application, especially in targeted therapy gene testing for CRC.

## Introduction

Colorectal Cancer, which includes both colon and rectal malignancies, is a common malignancy of the digestive tract. It is the third most common cancer worldwide, following lung and breast cancer, and is also the second leading cause of cancer-related deaths worldwide (1). Given the severity of CRC, early prevention, diagnosis, and treatment have become focal points of research (Buccafusca et al., 2019; Mauri et al., 2019) (2, 3). With the advancement of molecular biology techniques, there has been a deeper understanding of genetic alterations during CRC carcinogenesis. CRC is widely believed to be a complex disease involving multiple steps, stages, genes, and molecular regulations (4–6).

More than 90% of cancer-related deaths are attributed to distant metastases (7). As a critical link in the distant metastasis of malignant tumors, the presence and number of CTCs in a patient’s bloodstream not only reflects the ability of the primary tumor to invade blood vessels but also indicates the potential to form metastatic foci in distant organs (8). Therefore, the detection and quantification of CTCs is of significant prognostic value in assessing the risk of malignancy and metastasis of tumors. In addition, CTCs, as an integral part of liquid biopsy, can compensate for the inadequacy of traditional methods in the dynamic monitoring of tumor tissue and provide more precise personalized treatment guidance through real-time molecular and functional typing (9). However, traditional serological, imaging, and pathological testing methods struggle to detect CTCs, as only 1 CTC can be found per 10 billion normal blood cells, requiring an extremely sensitive detection method (10). Immunochemical magnetic nanoparticles can efficiently and selectively recognize and capture CTCs in whole blood (11). Currently, the only approved method for CTC detection is the CellSearch system, which uses EpCAM-coated to enrich CTCs and anti-CK antibodies for identification; however, this system has limitations, such as its inability to capture CTCs that have undergone epithelial−mesenchymal transition (EMT) (12, 13). Therefore, magnetic beads modified with other specific protein antibodies are required to achieve efficient enrichment of CTCs. A substantial body of research indicates that vimentin is highly expressed in various tumor cells, especially in tumor cells undergoing EMT (14, 15). Thus, vimentin may serve as a potential target for CTC capture in CRC.

High-throughput sequencing, also known as “next-generation” sequencing technology (NGS), is characterized by its ability to perform sequence determination of hundreds of thousands to millions of DNA molecules simultaneously, albeit with relatively short read lengths (16, 17). NGS methods fall into two main categories: mutational genomic panel sequencing of solid tumors and liquid biopsy of peripheral blood and urine (18, 19). Solid tumor NGS panels reflect only the status of the tumor at one point in time, which is very timely and necessary to guide the treatment of newly diagnosed patients. However, for patients who need to track the progression of tumor drug resistance throughout the process, the status of the tumor in the body will change over time (20, 21). In addition, for cancer patients who have already metastasized at the time of detection, taking a sample from only a certain part of the cancer tissue may not reflect the overall condition of the patient; for patients who have undergone surgery to reduce the tumor burden, rebiopsy of the tissue may be difficult. Therefore, there is a strong clinical need and scientific research value for NGS-based CTC liquid biopsy-targeted gene detection.

Molecular cytopathology, a growing specialty, offers insights into personalized therapy responses and prognoses from cytological neoplasm samples. Liquid biopsies, monitoring biomarkers like CTCs and ctDNA in blood and body fluids, are non-invasive and can be repeated, even in patients with comorbidities. Despite challenges posed by low biomarker concentrations, sensitive molecular techniques can detect them, necessitating validation and integration with tissue-based assessments (22).

This study aims to develop an efficient and specific CTC sorting system for CRC based on multifunctional targeted IMBs to achieve efficient, rapid, and accurate separation and enrichment of peripheral blood CTCs. By using NGS to detect hot mutation genes in patient tissues and Sanger sequencing to detect hot mutation genes in CTCs, we aimed to understand the consistency and differences between the tissue and peripheral blood levels of CRC and the trends of these differences. The aim of this study is to solve the clinical problem of difficult tumor tissue sampling and provide a reference for gene mutation detection in early diagnosis, efficacy evaluation, prognosis assessment, and micrometastasis detection in tumor patients.

## Materials and methods

### Sample collection and processing

The study included a total of 23 CRC patients treated at our hospital from January 2023 to July 2023. Patient selection was based on the availability of high-quality CTC samples and aimed to represent a diverse range of disease stages and characteristics. The 23 patients included in this study were carefully chosen to ensure that the sample population was representative of the broader patient cohort. Despite the relatively small sample size, the rigorous study design, including the use of validated markers and standardized CTC isolation and enumeration procedures, ensures the reliability and validity of the results obtained. A control group of 20 healthy volunteers was also recruited, and 7.5 mL of blood was collected from each volunteer. The inclusion criteria were as follows: (I) aged between 18 and 90 years; (II) had histologically confirmed CRC (AJCC stages I - IV); and (III) signed written informed consent before participation. The exclusion criteria were as follows: (I) unresectable primary or metastatic tumors; (II) any treatment received prior to surgery (including chemotherapy, radiotherapy and targeted therapy); and (III) other concurrent malignancies.

Peripheral blood samples of 15 mL were collected from CRC patients via medical blood collection tubes containing EDTA/K2 as an anticoagulant. The samples were stored at 4°C and protected from freezing during storage, processing and transport to ensure sample quality. All samples were assayed within 72 hours. This study’s adoption of a 72-hour timeframe is supported by several rationales: pre-experiments established the stability of CTC biomarkers and genetic material within this period, ensuring the impact on detection outcomes is negligible. Considering geographical disparities in sample collection and laboratory operational schedules, a 72-hour window is a feasible timeframe to process samples under optimal conditions. Rigorous quality control measures, such as the use of anticoagulant tubes and 4°C refrigeration, were employed to maintain sample integrity. Comparative analysis with samples processed within 24 hours verified the high consistency of results processed within 72 hours, validating our chosen protocol. The detection indicators included (1) counting CTCs in the peripheral blood of CRC patients via the magnetic separation immunofluorescence identification method; (2) analyzing hotspot mutated genes in the patient’s tumor tissue via next-generation sequencing (NGS); and (3) genetic testing of CRC CTCs.

### Experimental materials and instruments

In this study, we used the CRC cell lines CT26, HCT-8, SW480 and LS174T from the ATCC cell bank for our experiments. The cell culture conditions were strictly controlled in RPMI 1640 medium containing 10% neonatal calf serum, which was maintained at 37°C and 5% CO2 in an incubator. The key reagents and consumables used in the experiments were purchased from reputable biotechnology companies, including Solarbio’s Prussian blue staining kit, Gibco’s culture medium and serum, Abcam’s antibodies, Sigma’s DAPI staining solution, and eBioscience’s CD45-PE, among others. In addition, Huzhou Lie Yuan Medical Laboratory Co., Ltd. provided EpCAM antibody derivatives and magnetic nanoparticles, whereas Avanti supplied DSPC-APC. The high-end instruments and equipment used in the experiments included a BI-90Plus laser particle size analyzer/zeta potential analyzer from Bruker-Haven of America, an XL-30 environmental scanning electron microscope from Philips of the Netherlands, an LDJ9600-1 VSM magnetic property tester from Digital Instruments of America, an OLYMPUS B×61 fluorescence microscope from Olympus of Japan, and an XD-52AA rotary evaporator from Shanghai Banno Biotechnology Co., Ltd.

### Preparation of the IMB system

In this study, we innovatively employed the reverse evaporation method to prepare immunomagnetic beads (IMBs), marking a novel improvement upon existing techniques aimed at enhancing the efficiency and specificity of circulating tumor cell (CTC) separation. Our methodological contribution lies in the development of a new preparation technique for IMBs, which, to our knowledge, has not been previously reported. This advancement offers a new tool for the detection and analysis of CTCs. The experimental steps were as follows: First, 5 mg of 1,2-dioleoyl-sn-glycero-3-phosphocholine (DOPC) and 5 mg of cholesterol (Chol) were accurately weighed and added separately to two 50 mL three-necked flasks. A total of 1.0 mL of Fe3O4-HMN solution was measured, the ethanol was removed, the solution was dissolved in 3.0 mL of dichloromethane (CH2Cl2), and the mixture was transferred to the abovementioned three-necked flasks. The mixture was emulsified in an ice bath for 6 minutes via an ultrasonic probe processor. During this time, 2 mg of the EpCAM antibody 1,2-distearoyl-sn-glycero-3-phosphoethanolamine-N-[7-(dimethylamino)-4-trifluoromethyl]coumarin (GHDC) was dissolved in 6 mL of double-distilled water (ddH2O) and slowly injected into the three-necked flask. At the end of the ultrasonic treatment, the remaining CH2Cl2 was removed via a rotary evaporator. Finally, Ep-IMB was isolated via magnetic separation and triple-washed for purity. Vi-IMB was also synthesized successfully via the same method.

### Characterization testing of IMB systems

In this study, we used a BI-90Plus laser particle size analyzer/zeta potential analyzer to accurately measure the particle size and zeta potential of the IMBs. In addition, the microscopic morphology of the IMBs under different modification conditions was observed via atomic force microscopy (AFM). UV absorption spectroscopy was performed on the IMB solution via a UV−Vis spectrophotometer to analyze its spectral characteristics. We also used a Bio-Rad FTS3000 Fourier transform infrared (FTIR) spectrometer to observe and analyze the functional groups on the surface of the microspheres or modified materials. Finally, to verify the cell capture ability of the IMBs, we stained CT26 cells captured by the IMBs via a Prussian blue staining kit.

### Testing the cytotoxicity of the IMB system in CRC cell lines

In this study, we used standard cell culture techniques to culture CRC cell lines (including CT26, HCT-8, SW480 and LS174T). Specifically, the cells were cultured in complete RPMI 1640 medium supplemented with 10% fetal bovine serum (FBS) and 1% penicillin−streptomycin at 37°C and 5% CO2 in a humidified environment. To prepare a single-cell suspension, we digested the CRC cells with trypsin and then added the cell suspension to serum-containing medium to neutralize the trypsin. By diluting the cells and using a cell counting chamber, we accurately determined the cell concentration. The cells were then plated in a 96-well plate with 1000 cells per well, and 200 μL of culture medium was added to each well. After overnight incubation at 37°C, different concentrations of IMB were added to each well to yield final concentrations of 0, 10, 50, 100 and 200 μg/mL. The cells were then incubated for an additional 24 hours at 37°C, after which 10 μL of 5 mg/mL MTT reagent was added to each well, after which the mixture was returned to the incubator for 3 hours. After incubation, the medium was removed, and 200 μL of dimethyl sulfoxide (DMSO) was added to each well. Finally, a Spectra Max M5/M5e multifunctional microplate reader (Molecular Devices) was used to read the absorbance at a wavelength of 560 nm, which was used to analyze and process the experimental data.

### Measuring the cell capture efficiency of the IMB system

In this study, we first prepared a single-cell suspension of the CT26 cell line and accurately adjusted the cell concentration to 100 cells/7.5 mL to simulate the suspension state of CTCs. This simulated CTC suspension was subsequently divided equally into four experimental groups: the Ep-IMB group, the Vi-IMB group, the Ep/Vi-IMB group and the Ep+Vi-IMB group. In the Ep-IMB and Vi-IMB groups, immunomagnetic beads were added in volumes of 9, 15, 21, and 27 μL, respectively. In the Ep/Vi-IMB group, the two types of immunomagnetic beads were mixed at a 1:1 volume ratio, and 9, 15, 21, and 27 μL of the mixed immunomagnetic beads were added. In the Ep-IMB + Vi-IMB group, 6, 10, 14, or 18 μL of Ep-IMB or Vi-IMB immunomagnetic beads were added sequentially, with triplicate samples for each group. By comparing the capture efficiencies of each group, we aimed to determine the optimal capture scheme. After determining the best capture scheme, we applied it to capture experiments with CRC cells such as CT26, HCT-8, SW480, and LS174T cells. In the experiment, we added 10, 50, 100, 200, 500, and 1000 cells to 2 mL of phosphate buffer solution (PBS) and calculated the sensitivity of the capture scheme. To evaluate the specificity of the capture scheme, we also performed cell capture experiments using blood instead of PBS. In addition, we optimized the ratio of magnetic beads to antibodies in the immunomagnetic beads. In the experiment, we added 7 μL of IMBs containing 0, 10, 20, 30, 40, 50, 60, 70, or 80 μg of antibody and captured different numbers of CT26 cells, ultimately determining the best bead-to-antibody ratio according to the cell capture efficiency.

### Capture, characterization and enumeration of CTCs in blood samples from patients with CRC

In this study, we collected 7.5 mL peripheral blood samples from patients with CRC and stored them in anticoagulation tubes containing EDTA. The serum and plasma layers were then separated via centrifugation at 2500 rpm for 10 minutes and transferred to EP tubes. An equal amount of PBS was added to the EP tubes and mixed well. Next, 7 μL of Ep-IMB or Vi-IMB immunomagnetic beads were added sequentially to the tubes, which were subsequently incubated for 20 minutes at room temperature and mixed every 5 minutes. After incubation, the tubes were placed on a magnetic separation rack for 15 minutes, after which the waste solution was aspirated. To fix the cells, 10 μL of 4% paraformaldehyde solution was added, and the cells were fixed for 10 minutes. The cells were then washed three times with PBS. For immunofluorescence staining, 30 μL of DAPI staining solution, 10 μL of CK19-FITC staining solution and 10 μL of CD45-PE staining solution were added, mixed well and stained for 15 minutes under light-free conditions. After staining, the cells were washed three times with PBS. Finally, 15 μL of deionized water was added to the EP tubes to resuspend the cells, and then the cells were evenly spread on the anti-stick slides. After drying, the droplets were observed and counted under a fluorescence microscope.

### Tumor tissue and CTC nucleic acid extraction and mutation detection

In this study, we used the TIANamp Genomic DNA Kit to extract total DNA from tumor tissue and CTCs, ensuring that the total amount of tissue DNA exceeded 100 ng and that the total amount of CTC DNA exceeded 10 ng. The tumor tissue DNA samples were then sent to Huzhou Lie Yuan Medical Laboratory for high-throughput sequencing (NGS) via the Illumina NovaSeq 6000 platform to perform a comprehensive scan of 18 genes (including *KRAS*, *NRAS*, *BRAF*, *Tp53*, *APC*, *DPYD*, *NTRK*, *HER*-2, *UGT1A*, *PIK3CA*, *PTEN*, *POLD1*, *B2M*, *STK11*, *EGFR*, *MDM2*, *MDM4*, and *DNMT3A*). For CTC DNA, we followed the PCR procedures and systems described in **Supplementary Table S1** and **Supplementary Table S2** for amplification, using primers synthesized by Shanghai Bio-Engineering Co., Ltd., with specific sequence information provided in **Supplementary Table S3**. The amplified DNA fragments were then sent to Shanghai Bio-Engineering for sequencing to identify the gene mutation status.

High-throughput sequencing (NGS) and Sanger sequencing were utilized for their respective strengths in detecting genetic mutations. NGS’s high-throughput capability allows for the simultaneous sequencing of hundreds of thousands to millions of DNA molecules, making it ideal for analyzing multiple gene statuses and changes in tumor tissues. This comprehensive approach is vital for guiding treatment in newly diagnosed patients and tracking tumor evolution over time. Sanger sequencing, known for its precision, is employed for validating specific mutation genes in CTCs, which complements NGS by offering a detailed verification of suspected mutations. This combination leverages the strengths of both methods for a more accurate genetic analysis and personalized treatment guidance (Jennings, L. J., & Kirschmann, D. 2016, Schmid, K,eg al 2022) (23, 24).

### Statistical analysis

In this study, data analysis was performed via SPSS 21.0 statistical software. The experimental data are expressed as the means ± standard deviations (x ± s). One-way analysis of variance (ANOVA) was used for within-group comparisons. Statistical significance was determined according to the following criteria: when *P* < 0.05, the difference between the two groups was considered statistically significant and marked with an asterisk (*); when *P* < 0.01 or *P* < 0.001, the difference was considered highly significant and marked with two asterisks (**) and three asterisks (***), respectively.

## Results

### Preparation of immunoliposomal magnetic beads and CTC detection of CRC

**Figure 1:**
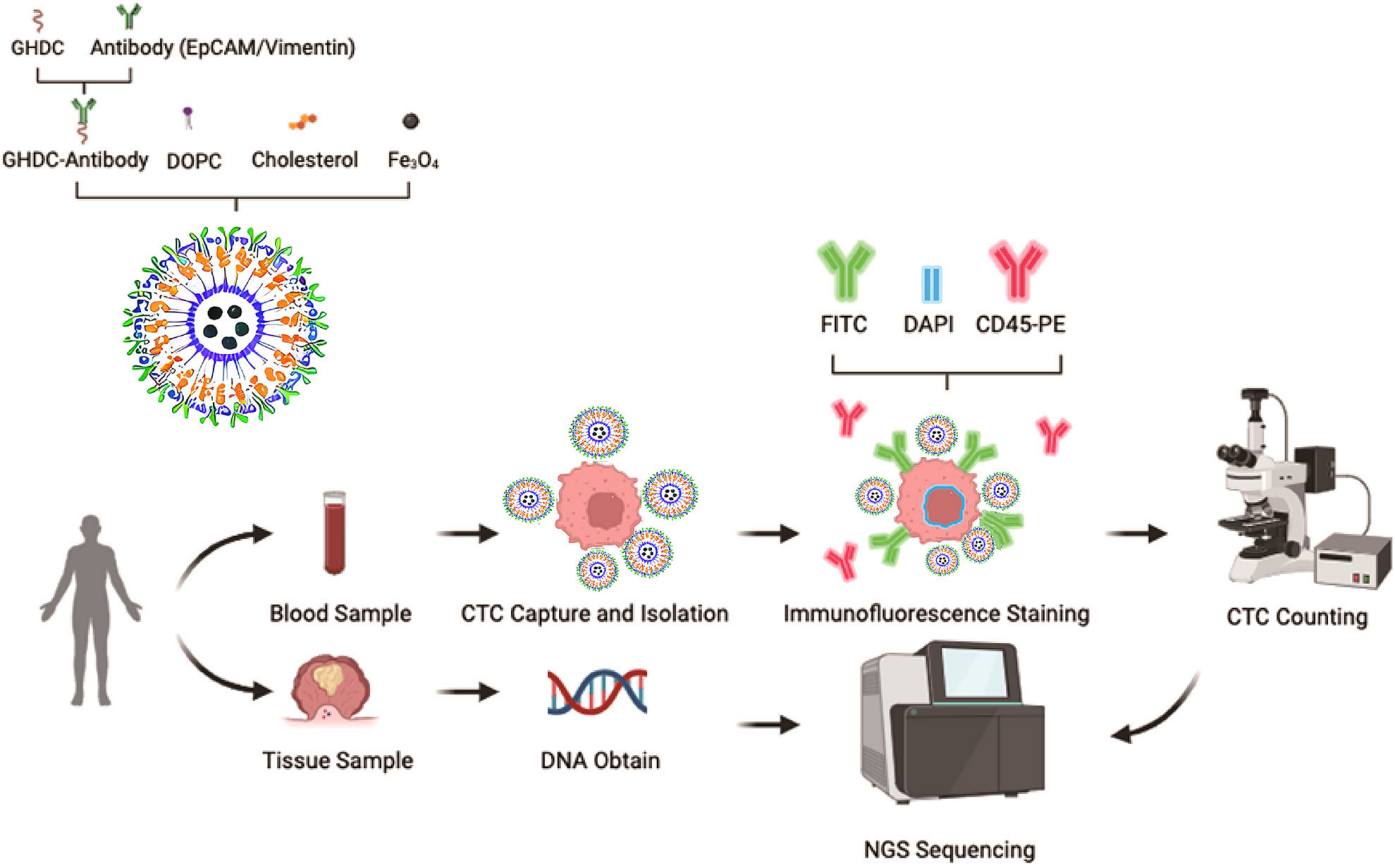
Flowchart of immunolipid magnetic ball (IMB) model preparation and CTC capture.

The IMBs were composed of five key components: (I) antibody derivatives designed for specific recognition of antigens on the surface of cancer cells; (II) iron oxide (Fe3O4) nanoparticles, which serve as the core of the beads to provide magnetic properties; (III) cholesterol, which enhances membrane stability; (IV) 1,2-dioleoyl-sn-glycero-3-phosphocholine (DOPC), a major component of the lipid bilayer; and (V) 1,2-dioleoyl-sn-glycero-3-phosphoethanolamine-N-[7-(diethylamino)-4-trifluoromethyl]coumarin (GHDC), which enhances fluorescent labeling. The integration of specific antibodies into the IMBs enabled highly selective capture of CRC CTCs. After capture, the CTCs were separated according to the magnetic properties of the superparamagnetic Fe3O4 nanoparticles and subsequently identified via immunofluorescence techniques. A schematic representation of multitarget immunomagnetic bead CTC detection is shown in **Figure 1**.

### Characterization and analysis of nanomagnetic beads

**Figure 2:**
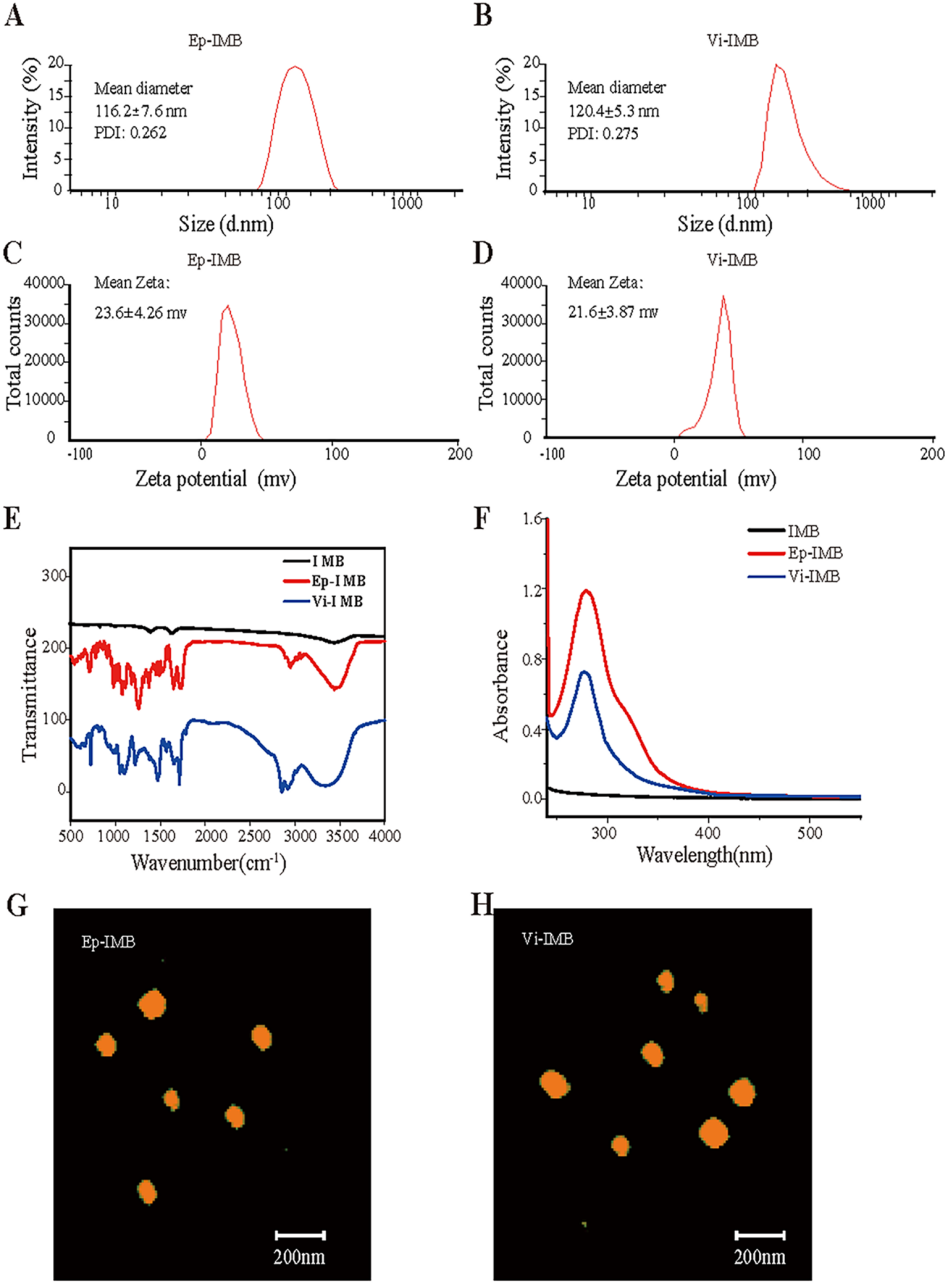
Characterization of the IMB models. **(A)** Ep-IMB particle size test plot; **(B)** Vi-IMB particle size test plot; **(C)** Ep-IMB potential test plot; **(D)** Vi-IMB potential test plot; **(E)** Immunolipid magnetic sphere infrared spectra; **(F)** Immunolipid magnetic sphere ultraviolet absorption spectra; **(G)** Ep-IMB AFM observation plot; **(H)** Vi-IMB AFM observation plot.

To further confirm that the manufactured magnetic beads had a small particle size and favorable stability, we analyzed the particle size and zeta potential of Ep-IMB and Vi-IMB. The average particle size of Ep-IMB was determined to be 116.2 nm (**Figure 2A**), with a zeta potential of +23.6 mV (**Figure 2B**). For Vi-IMB, the average particle size was measured to be 120.4 nm (**Figure 2C**), and the zeta potential was +21.6 mV (**Figure 2D**).

Infrared spectroscopy analysis (**Figure 2E**) revealed characteristic absorption peaks for Ep-IMB and Vi-IMB at 1725 cm^-1^ for the ester carbonyl (C=O) stretching vibration, 1186 cm^-1^ for the ether (C-O-C) stretching vibration, 1650 cm^-1^ for the amide carbonyl (C=O) stretching vibration, and 3500 cm^-1^ for the amine (C-N) stretching vibration. These characteristic peaks confirmed the successful synthesis of the IMBs.

Ultraviolet spectroscopy (**Figure 2F**) revealed a broad absorption peak at approximately 279 nm for both types of antibody-modified magnetic beads, indicating that EpCAM and vimentin were successfully conjugated to the surface of the beads.

The AFM images (**Figures 2G, H**) revealed that both types of immunomagnetic microspheres were spherical in shape, varied in size, and did not aggregate, indicating their good stability and regular morphology. The size ranged from 100 to 150 nm, showing characteristics of liposome-like vesicles.

### IML cytotoxicity and capture efficiency

**Figure 3:**
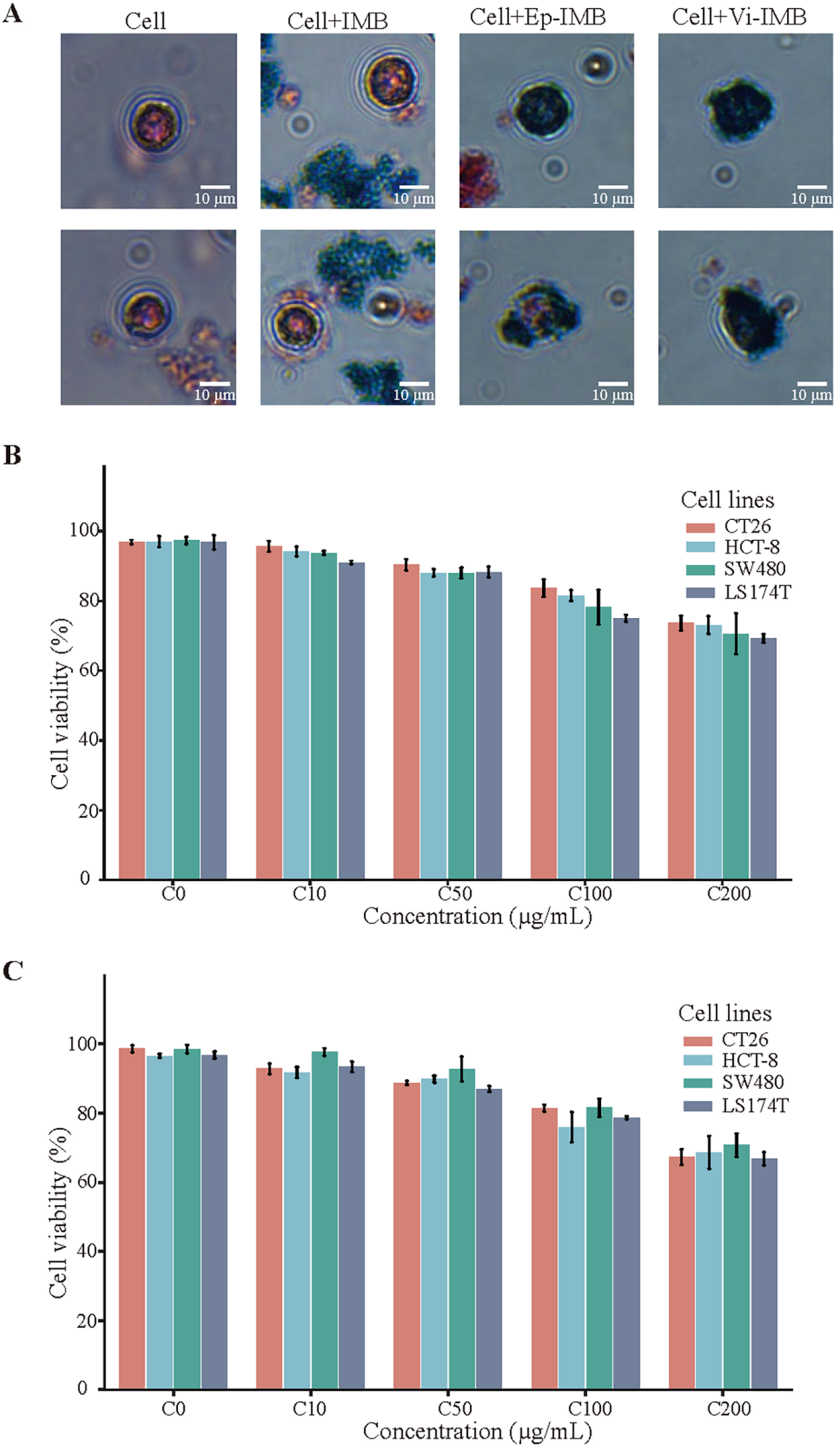
Cytotoxicity assay of the IMB models. **(A)** Prussian staining results; **(B)** Cellular activity assay of Ep-IMB in cell lines (CT26, HCT-8, SW480, and LS174T); **(C)** Cellular activity assay of Vi-IMB in cell lines (CT26, HCT-8, SW480, and LS174T).

The Prussian blue staining results shown in **Figure 3A** clearly demonstrated robust cell growth, with cells exhibiting a typical regular morphology. The IMBs were randomly distributed around the cells, whereas Ep-IMB and Vi-IMB were uniformly attached to the cell surface, indicating the immune recognition properties of Ep-IMB and Vi-IMB. These findings were consistent with the results of the UV assays and did not adversely affect cell morphology. A closer examination of the data in **Figures 3B, C** revealed that Ep-IMB and Vi-IMB at a concentration of 50 μg/mL exhibited low toxicity toward various cancer cell lines, with cell viability rates above 90%. As the concentration of magnetic beads increased, the cell viability rate gradually decreased, indicating that high concentrations of beads have some inhibitory effect on cell growth. Even at a relatively high concentration of 200 μg/mL, the viability of the cell lines remained above 60%, suggesting that although Ep-IMB and Vi-IMB have a certain degree of cytotoxicity toward the cell lines, the overall level of toxicity was relatively low.

**Figure 4:**
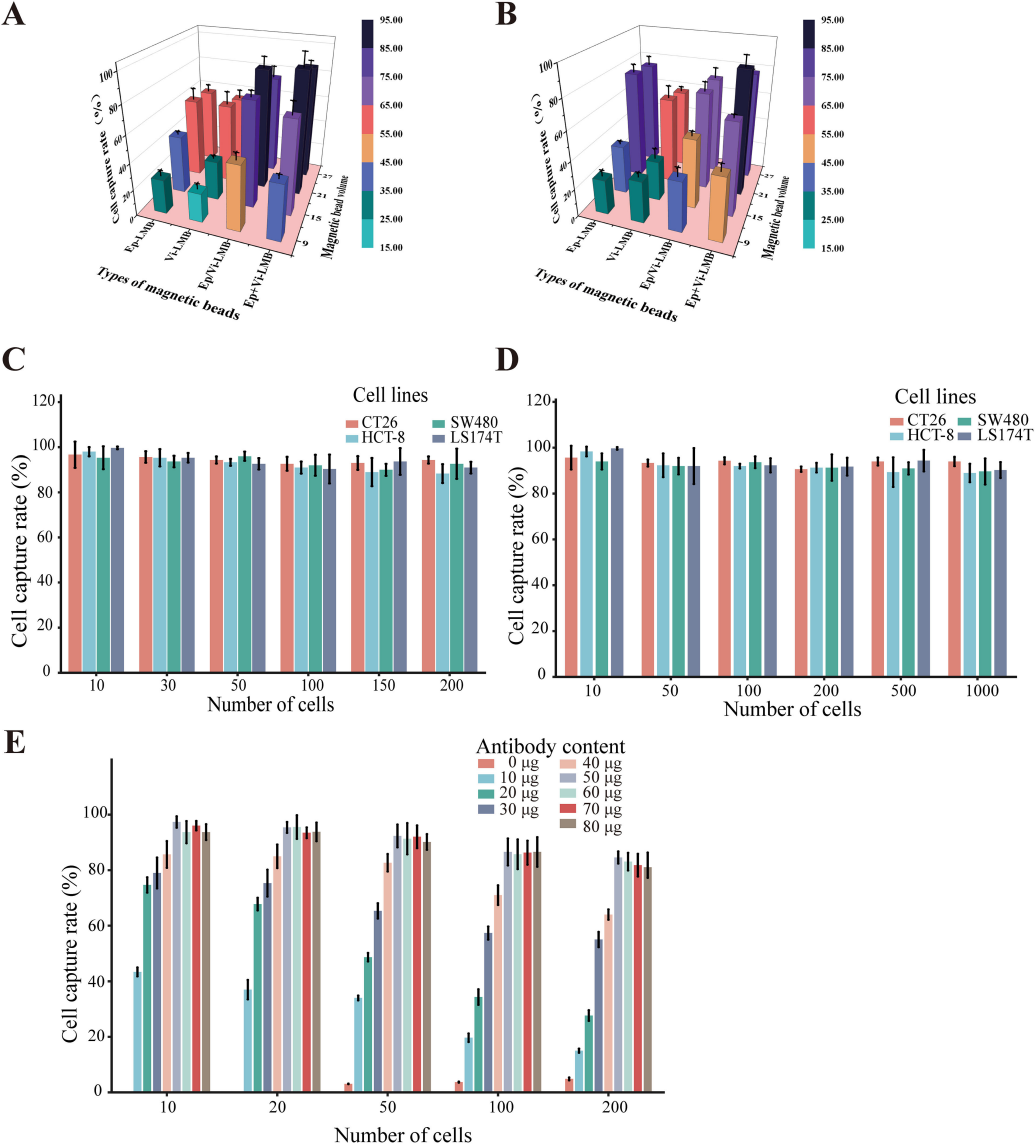
Cell capture efficiency of the IMB model. **(A)** Capture efficiency of CT26 cells by different inputs of the IMB model in PBS; **(B)** capture efficiency of CT26 cells by different inputs of the IMB model in the blood simulation system; **(C)** verification of the specificity of the IMB model in the PBS system; **(D)** verification of the sensitivity of the IMB model in the simulated blood system; **(E)** capture efficiency of CT26 cells by magnetic spheres prepared with different antibody contents in prepared magnetic spheres on the capture efficiency of CT26 cells. (Error lines represent the standard deviation).

To further validate the effects of different capture schemes and magnetic bead dosages on the efficiency of CRC cell capture, an experiment was conducted. The results showed that within the PBS system, the sequential introduction of Ep-IMB and Vi-IMB resulted in the highest capture efficiency when the magnetic bead dosage was held constant (**Figure 4A**). Notably, the capture efficiency peaked when 7 μL of Ep/Vi-IMB was added, and further increases in the magnetic bead dosage stabilized the capture efficiency. On the basis of these results, the sequential capture method with the addition of 7 μL of Ep/Vi-IMB was identified as the optimal strategy, which was subsequently verified in a simulated blood system (**Figure 4B**). In the PBS system, the average capture efficiency was evaluated across different cell lines and with a gradient of cell numbers, and the results demonstrated that the specificity of the capture system reached 90.54% (**Figure 4C**). In addition, the average capture efficiency tested in the simulated blood system indicated that the sensitivity of the separation system was 89.07% (**Figure 4D**). Furthermore, the cell capture efficiency was optimized by adjusting the ratio of magnetic beads to antibodies, with magnetic bead preparation being most effective at an antibody concentration of 50 μg (**Figure 4E**).

### Statistics on the sorting effect of various magnetic balls on CRC cells

A total of 23 patients with CRC were enrolled in this study, with a mean age of 62.7 years and a standard deviation of 16.5 years. There were 7 male patients, accounting for 30.4% of the total. The distribution of pathological stages from I to IV was relatively uniform, with proportions of 8.7%, 21.7%, 34.8%, and 34.8%, respectively. The main tumor sites were the left colon (21.7%) and the right colon (43.5%), with 4 cases in the rectum, 3 in the sigmoid colon and 1 in the transverse colon. Moderate differentiation was the predominant type, accounting for 65.2% of the cases. In terms of metastatic status, 8 patients had distant metastases, representing 34.8% of the total patients. More detailed information can be found in **Table 1**.

**Table 1:** Characteristics of the enrolled CRC patients.

Characterization	Number (%)
Age	62.7±16.5
Sex	
Female	16 (69.6)
Male	7 (30.4)
Pathological staging	
I	2 (8.7)
II	5 (21.7)
III	8 (34.8)
IV	8 (34.8)
Tumor site	
Left hemicolon	5 (21.7)
Right hemicolon	10 (43.5)
Rectum	4 (17.4)
Sigmoid colon	3 (13.0)
Transverse colon	1 (4.3)
Degree of differentiation	
Moderately differentiated	15 (65.2)
Moderately to poorly differentiated	4 (17.4)
Poorly differentiated	4 (17.4)
Distant metastatic site	
M0	15 (65.2)
M1	8 (34.8)

**Figure 5:**
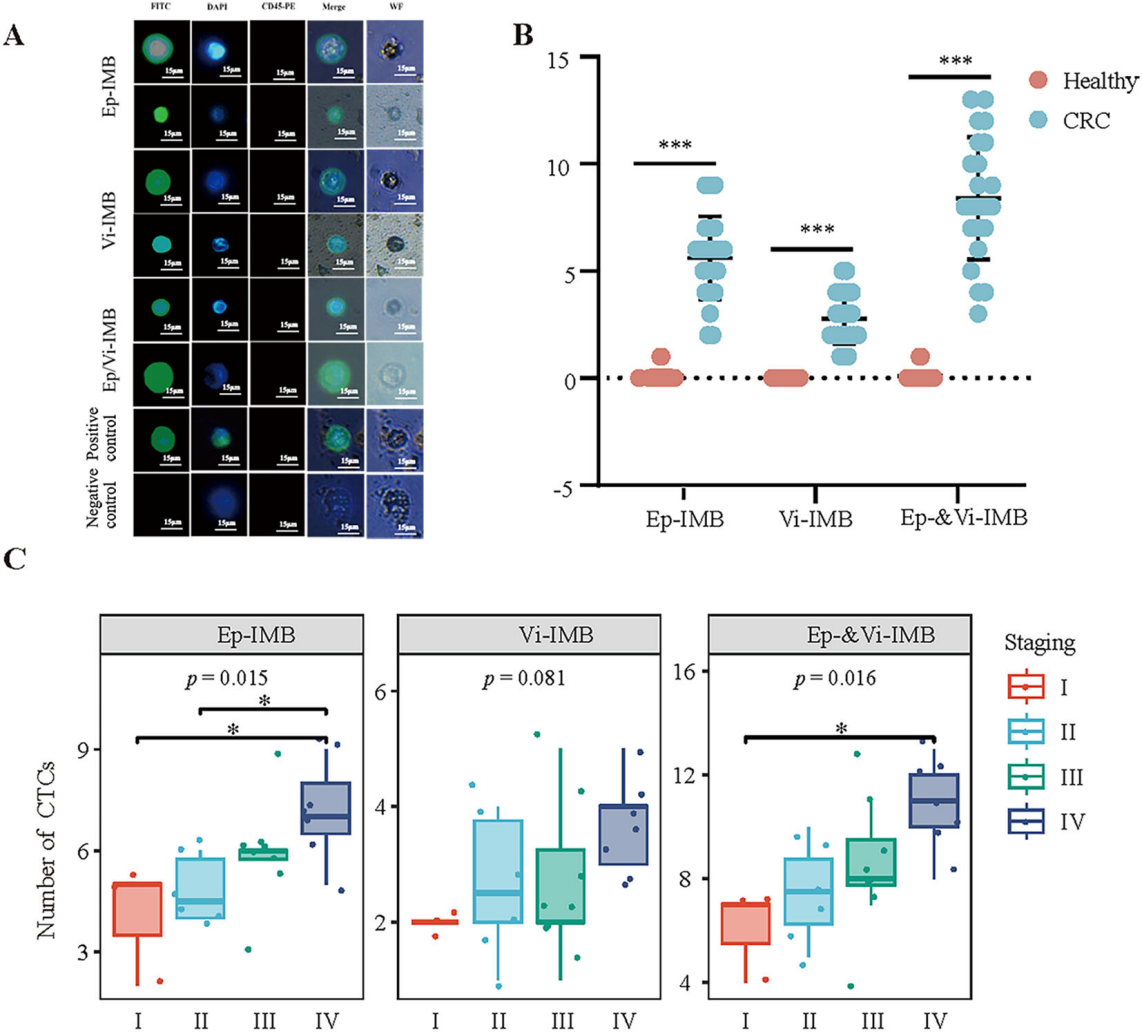
Immunofluorescence identification and counting of CTCs in blood samples from CRC patients. **(A)** Immunofluorescence identification of captured CTCs. **(B)** Comparison of the ability of the Ep-IMB, Vi-IMB and EP/Vi-IMB models to capture CTCs in the blood of CRC patients with the blood of the normal population. **(C)** The number of CTCs captured by the Ep-IMB, Vi-IMB and EP/Vi-IMB models in the blood of CRC patients with different pathological stages.

In the present study, we identified and quantified CTCs via immunofluorescence analysis. Under white light, the cellular morphology was clearly visible, with CK19-FITC fluorescence showing a strong positive signal in green and DAPI fluorescence showing a strong positive signal in blue, while CD45 staining was negative. These characteristics facilitated the accurate detection of CTCs (**Figure 5A**). Using these criteria, we performed CTC enumeration and statistical analysis on blood samples from 20 healthy individuals and 23 patients with CRC (**Figure 5B**).

The results showed that in patients with CRC, the average number of CTCs captured by Ep-IMB was 5.61 per 7.5 mL; the average number captured by Vi-IMB was 2.78 per 7.5 mL; and with the Ep/Vi-IMB system, we achieved an average CTC capture yield of 8.39 per 7.5 mL. In the blood samples from healthy individuals, a low number of CTCs was detected, with an average of 0.09 per 7.5 mL, and the CTC counts from all three capture systems were significantly lower than those from patients with CRC (*P* < 0.001).

Furthermore, we observed that the number of captured epithelial or mesenchymal CTCs gradually increased with increasing tumor stage, and the CTC counts from all three capture systems significantly differed across stages (**Figure 5C**). In addition, the number of CTCs captured by Ep-IMB and Vi-IMB differed significantly between stages II, III and IV (*P* < 0.05) (**Supplementary Figure S1**).

### Comparative analysis of genomic heterogeneity in CRC tissue and CTCs

**Figure 6:**
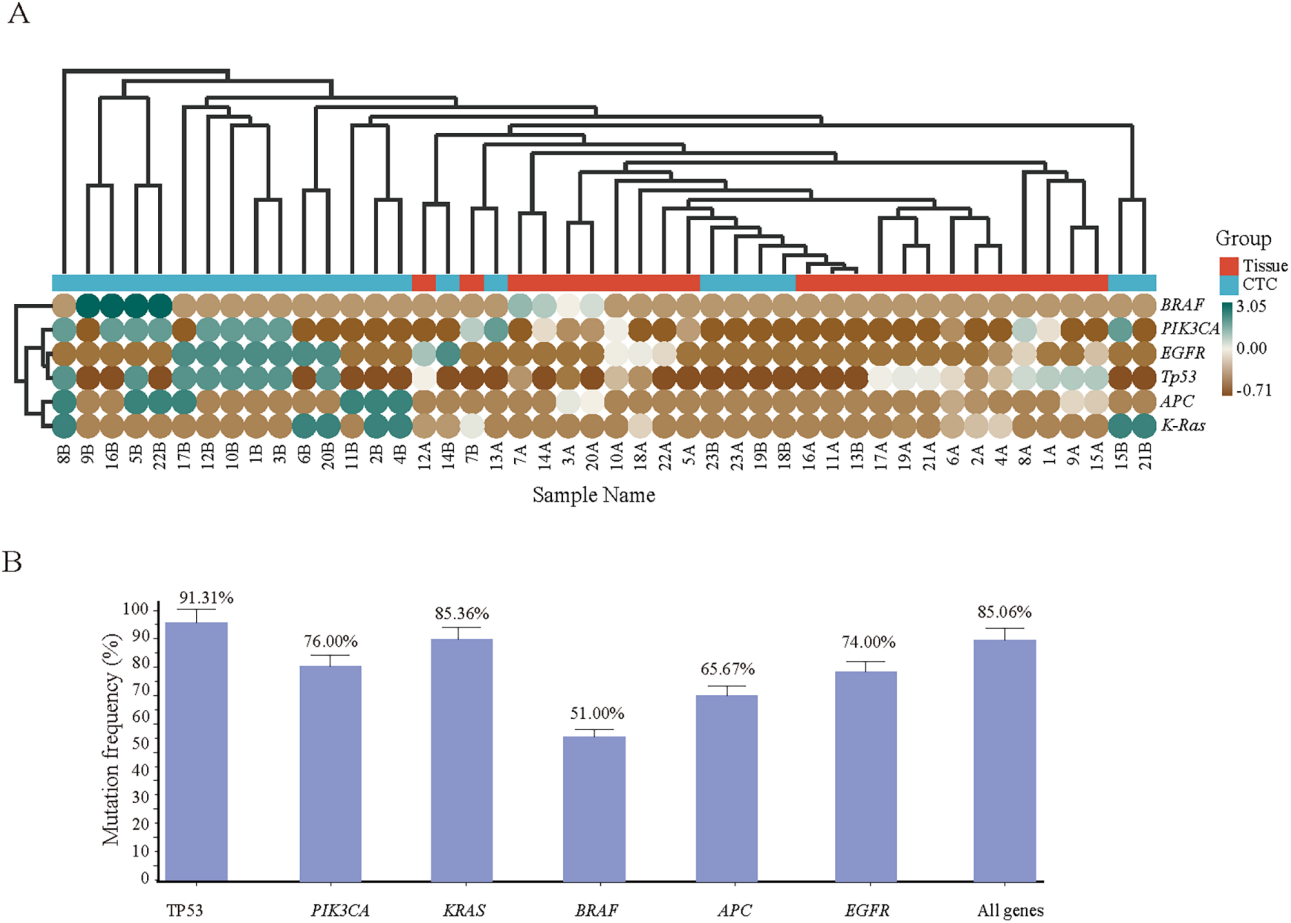
**(A)** onsistency validation of the tissue and corresponding CTC gene mutation results of 23 CRC patients. Hotspot gene mutation results of tissue and blood CTC samples from 23 CRC patients; **(B)** Consistency rate statistics of tissue and blood CTC gene mutations.

According to the heatmap, the *Tp53* gene mutation frequency in CRC tissue samples was the highest at 65.22%, followed by the *PIK3CA* (39.13%), KRAS (30.43%), *BRAF* (17.39%), *APC* (26.09%) and *EGFR* (34.78%) genes (**Figure 6A**). Subsequent testing of CTC samples by Sanger sequencing revealed that the Tp53 gene mutation showed the highest concordance (91.31%) compared with the hotspot gene mutations identified in tissue samples, whereas mutations in *PIK3CA* (76.00%), *KRAS* (85.36%), *BRAF* (51.00%), *APC* (65.67%) and *EGFR* (74.00%) also showed good concordance. Overall, the two detection methods achieved a concordance rate of 85.06% in terms of genetic mutation (**Figure 6B**).

## Discussion

Liquid biopsy has emerged as a burgeoning focal point within the realm of oncological research, demonstrating substantial potential across various stages of cancer screening and therapeutics. In asymptomatic populations, this noninvasive approach can identify individuals harboring cancer, thereby increasing early detection rates and facilitating more efficacious intervention strategies. Moreover, it provides dynamic insights into the progression of CRC and assesses the sensitivity of the disease to therapeutic interventions (25). CTCs have played a critical role in the advancement of precision medicine. Compared with traditional invasive tissue biopsies and cytology, CTC detection offers several distinct advantages, including a straightforward sample collection process and the ability to perform multiple repeated assessments (26). As intact neoplastic cells, CTCs provide a wealth of information on the genomic, transcriptomic, proteomic and metabolomic profiles of tumors (27). In addition, CTCs can be analyzed ex vivo, making it easier to study tumor cells (28).

In 2010, the American Joint Committee on Cancer (AJCC) included CTCs in the TNM cancer staging system for the first time (29). The study of CTC dynamics throughout tumor progression offers potential predictive information for cancer screening, prognostic assessment, disease monitoring and therapeutic response. Currently, CTCs have been extensively used in the prognostic evaluation of various malignancies, including lung, head and neck, esophageal, gastric and breast cancer (30–33). Given the low five-year survival rate for patients with advanced colorectal cancer, early detection and treatment are critical for improving survival. Yang et al. (34) used an immunoaffinity negative enrichment method to detect CTCs in benign colorectal disease patients (such as polyps) and nonmetastatic CRC patients preoperatively and reported that the CTC count in CRC patients was significantly greater than that in patients with colorectal polyps (3.47 ± 0.32 cells/3.2 mL vs. 1.49 ± 0.2 cells/3.2 mL, *P* < 0.001). Tsai et al. (35)also demonstrated a close correlation between CTC counts and disease progression (*P* < 0.0001), with high specificity (86%) and sensitivity (79%) in 95% of CRC stages and 79% of adenomatous lesions. Sastre et al. (36) used the CellSearch system to detect peripheral blood CTCs in 1202 CRC patients and reported that a CTC count of ≥3 cells/7.5 mL was associated with adverse prognostic factors. The CellSearch system was once an option on the domestic market, it was discontinued in 2016 because of its low detection rate. Although Parsortix device from Angle Company has been FDA-approved to enumerate and sort CTCs, providing an alternative technology in this field, Recently. In this study, a novel CTC sorting system was developed on the basis of the CellSearch system, which sequentially enriches CTCs with Ep/Vi-IMB and demonstrates good dispersion, stability and low cytotoxicity, with a specificity of 90.54% and a sensitivity of 89.07%. Therefore, our research retains its uniqueness and value. Our IMB system, by combining EpCAM and vimentin dual markers, effectively captures CTCs, including those undergone EMT, demonstrating higher specificity and sensitivity compared to the CellSearch system. To address the potential for false positives associated with vimentin as a marker, we implemented a dual-marker approach using both EpCAM and vimentin-modified immunomagnetic beads (Ep-IMBs and Vi-IMBs) for the capture of CTCs. This strategy ensures that only cells co-expressing both markers are classified as CTCs, thereby increasing specificity. We also utilized highly specific vimentin antibodies to reduce non-specific binding, which was optimized through preliminary experiments and competitive assays. The inclusion of additional unique tumor-associated antigens in our detection system further enhances specificity by capitalizing on the distinctive features of CTCs. Finally, stringent data analysis, including the exclusion of non-tumor cells by negating common leukocyte markers such as CD45, was performed to minimize false positives. Compared to microfluidic and nanoparticle-based technologies, the IMB system offers advantages in cost-effectiveness and ease of operation while maintaining low cytotoxicity, which is crucial for clinical applications (37, 38). Nevertheless, the preparation process of the IMB system is relatively complex, and future studies should focus on further optimization to enhance its practicality and clinical feasibility. The average detection volume of CTCs in the blood of CRC patients was 8.39 cells/7.5 mL. Notably, the detection rate of CTCs increased with the TNM stage of the patients, indicating a close relationship between the number of CTCs and the progression of primary malignancies and suggesting its potential as an adjunct indicator for CRC TNM staging. However, the relationships between the number and type of CTCs and specific pathological histological characteristics and actual degree of tissue metastasis require further in-depth research.

Precision medicine has achieved significant success in the treatment of CRC, with the key being the detection of specific mutated genes to select the most appropriate targeted therapeutic agents. Compared with traditional detection methods, next-generation sequencing (NGS) technology can analyze the status and alterations of multiple genes in the body in a single high-throughput test, greatly enriching the clinical applications for CRC patients in diagnosis, treatment, and disease prognosis monitoring. In an NGS study of 526 CRC tumor samples, mutations in the *TP53* and *KRAS* genes were found to be frequent, whereas mutations in *PIK3CA* often coexisted with *KRAS*, *NRAS*, or *BRAF* mutations (39). The *TP53* gene encodes the p53 protein, an important tumor suppressor, and any mutation that inactivates this protein can lead to tumorigenesis (40, 41). Mutation of the *KRAS* gene, a member of the RAS family, can lead to continued activation of the RAS protein, thereby causing abnormal cell growth and proliferation and promoting tumor development (42, 43). This study uses next-generation sequencing (NGS) technology to analyze colorectal tumor tissue and reveals high mutation frequencies in the *Tp53*, *PIK3CA*, *EGFR*, and *KRAS* genes. Subsequently, when patient-isolated CTCs were used as templates, hotspot mutation genes were verified via Sanger sequencing, and the mutation rate and its consistency with tissue sample mutations were calculated. The study showed high consistency for mutations in *Tp53* (91.31%), *PIK3CA* (76.00%), *KRAS* (85.36%), *BRAF* (51.00%), *APC* (65.67%), and *EGFR* (74.00%), with an overall gene mutation consistency rate of 85.06%. This finding indicates that there is a high degree of concordance between the tumor-related hotspot mutation genes detected by NGS technology in CRC tissue samples and those detected by Sanger sequencing in CTC samples. Therefore, in cases where it is difficult to obtain tumor tissue samples, enriched CTCs may serve as a suitable alternative for clinically targeted drug gene mutation detection, effectively overcoming the challenge of tumor tissue sampling.

## Limitations of the study

This study focused on several key hotspot mutated genes, but given the complexity of the tumor genome, future studies will need to use advanced means such as whole-genome sequencing to achieve a more comprehensive analysis of genetic variants. In addition, although this study proposed an individualized treatment strategy based on CTCs, its applicability and efficacy in different patient groups still need to be validated in large-scale clinical trials. Moreover, this study lacked data on long-term patient tracking and follow-up, which limited the in-depth understanding of CTCs as indicators for prognosis and efficacy assessment. Therefore, future studies should include long-term clinical follow-up to assess the role of CTCs in cancer treatment more comprehensively.

## Conclusion

The analysis of CTCs offers us a crucial tool for comprehending and capitalizing on tumor heterogeneity, which facilitates a more profound understanding of the biological properties of tumors and guides the development of personalized therapeutic strategies. Future studies will delve deeper into the role of CTCs in tumor heterogeneity, using this knowledge to refine treatment plans and potentially increase patient survival rates.

Building on previous studies that enhanced the capture efficiency of circulating tumor cells (CTCs) using immunolipid magnetic bead (IMB) systems modified with specific antibodies (44, 45), our research has achieved significant technological advancements: Firstly, we employed a reverse evaporation method for IMB preparation and optimized the ratio of EpCAM to Vimentin antibodies, thereby enhancing the uniformity and stability of CTC capture. Secondly, we validated the IMB system in a larger cohort of 23 colorectal cancer (CRC) patients, yielding more compelling data on clinical relevance. Additionally, we analyzed gene mutations in CTCs using next-generation sequencing (NGS) and Sanger sequencing, finding a high degree of concordance with mutations in tumor tissues. Lastly, we addressed the heterogeneity of CTCs by employing a dual-marker strategy with EpCAM and Vimentin, allowing for a comprehensive assessment of CTC biology. In summary, we have successfully developed a CTC sorting system based on Ep-IMB and Vi-IMB technology that efficiently captures CTCs from the peripheral blood of CRC patients and detects clinically relevant genetic mutations, effectively overcoming the challenge of obtaining tumor tissue samples. This system not only offers a novel approach for early diagnosis, therapeutic efficacy evaluation, prognostic assessment, and minimal residual disease detection but also holds significant potential for clinical application in the field of targeted therapy gene testing for CRC.

## Data Availability

The original contributions presented in the study are includedin the article/**Supplementary Material**. Further inquiries can be directed to the corresponding author/s.

## Supplementary material

Supplementary Figure 1 Differences in the number of CTCs captured by Ep-IMB and Vi-IMB among different pathological stages.
