# Supplementary figures and images for: Immunolipid magnetic bead-based circulating tumor cell sorting: a novel approach for pathological staging of colorectal cancer
Source: Front Oncol. 2025 Jan 24;14:1531972. doi: 10.3389/fonc.2024.1531972 (PMC11803635; doi:10.3389/fonc.2024.1531972)

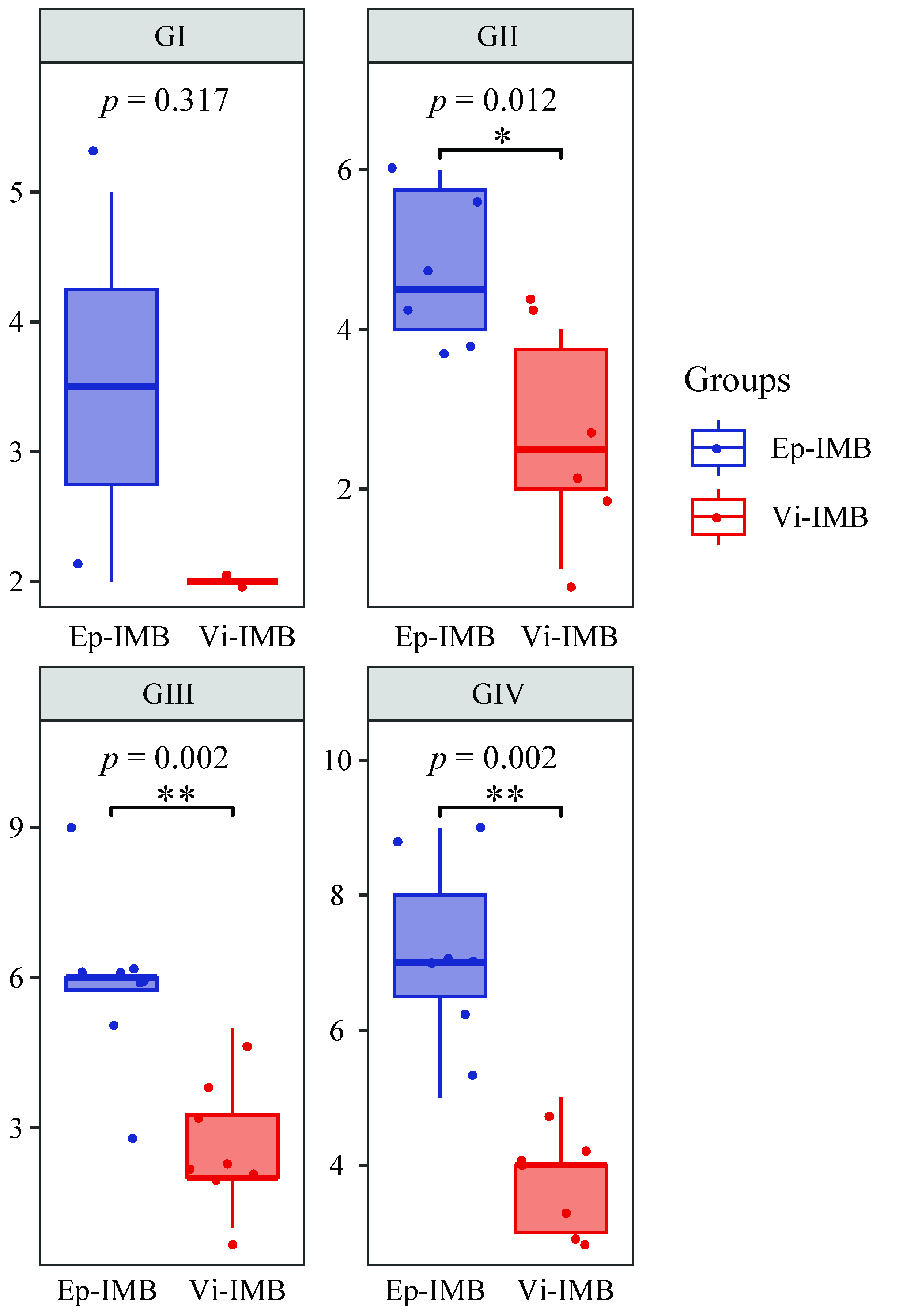

Supplement: Supplementary Figure 1 — Differences in the number of CTCs captured by Ep-IMB and Vi-IMB among different pathological stages. [file Image1.tif]
