# Supplementary material for: Immunolipid magnetic bead-based circulating tumor cell sorting: a novel approach for pathological staging of colorectal cancer
Source: Front Oncol. 2025 Jan 24;14:1531972. doi: 10.3389/fonc.2024.1531972 (PMC11803635; doi:10.3389/fonc.2024.1531972)
Supplement: Supplementary file 2 [file Table1.docx]

**Supplementary Table 1** PCR reaction parameters

| process | Temperature (℃) | Time (s) | Number of cycles |
| --- | --- | --- | --- |
| Pre-denaturation | 94 | 180 | 1 |
| Denaturation | 94 | 15 | 35 |
| Annealing | 60 | 20 |  |
| Extension | 72 | 60 |  |
| Final Extension | 72 | 300 | 1 |
| Preservation | 4 | ∞ |  |
