# Supplementary material for: Immunolipid magnetic bead-based circulating tumor cell sorting: a novel approach for pathological staging of colorectal cancer
Source: Front Oncol. 2025 Jan 24;14:1531972. doi: 10.3389/fonc.2024.1531972 (PMC11803635; doi:10.3389/fonc.2024.1531972)
Supplement: Supplementary file 3 [file Table2.docx]

**Supplementary Table 2** PCR reaction system

| System composition | Dosage (μL) |
| --- | --- |
| 2×Taq MasterMix（Dye） | 15 |
| Upstream Primers | 0.75 |
| Downstream Primers | 0.75 |
| DNA products | 1.5 |
| ddH_2_O | up to 30 |
