# Supplementary material for: Immunolipid magnetic bead-based circulating tumor cell sorting: a novel approach for pathological staging of colorectal cancer
Source: Front Oncol. 2025 Jan 24;14:1531972. doi: 10.3389/fonc.2024.1531972 (PMC11803635; doi:10.3389/fonc.2024.1531972)
Supplement: Supplementary file 4 [file Table3.docx]

**Supplementary Table 3** PCR primer sequences

| Gene | Primer sequence (5'→3') |
| --- | --- |
| Tp53-Exon5-F | 5′- TTTGCCAACTGGCCAAGACC-3′ |
| Tp53-Exon5-R | 5′-ACAACCTCCGTCATGTGCTG-3′ |
| PIK3CA-Exon10-F | 5′- GTAACAGACTAGCTAGAGACAATGA-3′ |
| PIK3CA-Exon10-R | 5′-TTCTCCTGCTCAGTGATTTCAGA-3′ |
| K-Ras-Exon2-F | 5′-TAAACTTGTGGTAGTTGGAGCTG-3′ |
| K-Ras-Exon2-R | 5′-CTCTATTGTTGGATCATATTCGTCC-3′ |
| BRAF-Exon15-F | 5′-ACACGCCAAGTCAATCATCCAC-3′ |
| BRAF-Exon15-R | 5′-ACATCTGACTGAAAGCTGTATGGAT-3′ |
| APC-Exon16-F | 5′-TCCTAGAGGAGCCAAGCCAT-3′ |
| EGFR-Exon19-F | 5′-GCCTAGACGCAGCATCATTA-3′ |
| EGFR-Exon19-R | 5′-ATGCCTCCATTTCTTCATCC-3′ |
